# Walking with a powered ankle-foot orthosis: the effects of actuation timing and stiffness level on healthy users

**DOI:** 10.1186/s12984-020-00723-0

**Published:** 2020-07-17

**Authors:** Marta Moltedo, Tomislav Baček, Ben Serrien, Kevin Langlois, Bram Vanderborght, Dirk Lefeber, Carlos Rodriguez-Guerrero

**Affiliations:** 1 Department of Mechanical Engineering, R&MM Research Group, and Flanders Make, Vrije Universiteit Brussel (VUB), Pleinlaan 2, Brussels, 1050 Belgium; 2 Department of Biomechanics, Vrije Universiteit Brussel (VUB), Pleinlaan 2, Brussels, 1050 Belgium

**Keywords:** Powered ankle-foot orthosis, Variable stiffness actuator, Robotics, Exoskeleton, Gait

## Abstract

**Background:**

In the last decades, several powered ankle-foot orthoses have been developed to assist the ankle joint of their users during walking. Recent studies have shown that the effects of the assistance provided by powered ankle-foot orthoses depend on the assistive profile. In compliant actuators, the stiffness level influences the actuator’s performance. However, the effects of this parameter on the users has not been yet evaluated. The goal of this study is to assess the effects of the assistance provided by a variable stiffness ankle actuator on healthy young users. More specifically, the effect of different onset times of the push-off torque and different actuator’s stiffness levels has been investigated.

**Methods:**

Eight healthy subjects walked with a unilateral powered ankle-foot orthosis in several assisted walking trials. The powered orthosis was actuated in the sagittal plane by a variable stiffness actuator. During the assisted walking trials, three different onset times of the push-off assistance and three different actuator’s stiffness levels were used. The metabolic cost of walking, lower limb muscles activation, joint kinematics, and gait parameters measured during different assisted walking trials were compared to the ones measured during normal walking and walking with the powered orthosis not providing assistance.

**Results:**

This study found trends for more compliant settings of the ankle actuator resulting in bigger reductions of the metabolic cost of walking and soleus muscle activation in the stance phase during assisted walking as compared to the unassisted walking trial. In addition to this, the study found that, among the tested onset times, the earlier ones showed a trend for bigger reductions of the activation of the soleus muscle during stance, while the later ones led to a bigger reduction in the metabolic cost of walking in the assisted walking trials as compared to the unassisted condition.

**Conclusions:**

This study presents a first attempt to show that, together with the assistive torque profile, also the stiffness level of a compliant ankle actuator can influence the assistive performance of a powered ankle-foot orthosis.

## Background

Powered ankle-foot orthoses (PAFOs) are robotic devices meant to assist the ankle joint of their users. Recently, several PAFOs have been developed and tested to investigate their potential in reducing the biological effort of healthy users during assisted walking as compared to unassisted or normal walking [1–5], but also to evaluate their performance when used as assistive or rehabilitation devices with weakened users such as the elderly [6, 7] and impaired subjects [8–10]. In these studies, PAFOs where shown to be able to reduce the metabolic cost of walking of healthy users as compared to the unassisted configuration [1, 5, 11], and in some cases also as compared to walking without the device [3, 4]. Furthermore, studies with impaired subjects showed the advantages of using a PAFO to improve the impaired ankle kinematics, to increase the walking speed of the assisted subject, and to improve the gait symmetry [8, 9, 12, 13]. Despite the promising results obtained by these studies, when comparing the performance of different PAFOs in achieving similar goals, differences exist in the outcomes of these experiments. As reported in [13], some studies have assessed the time needed by the users to adapt to the assistance provided by PAFOs. Although, several of these studies have shown that, over different identical sessions, the subjects reached a steady state increasingly faster [5, 11, 14–17], the resulting adaptation times differ between studies. In addition to this, different studies found divergent outcomes regarding the capabilities of PAFOs in reducing the metabolic cost and muscle activation of healthy users during powered walking [11, 14, 18–20]. The divergences come from the influence that some parameters of the assistive profile provided by the PAFO have on the user [1, 3, 13, 21, 22]. Recently, several research groups have evaluated the effects of two of these assistive parameters, which are the push-off onset time [1, 3, 21, 23] and the plantarflexion assistance magnitude [1, 22, 24, 25]. Specifically, these studies assessed the effect that different values of these parameters have on the biological effort of healthy users during walking. The results of these studies were analyzed and compared in [13]. This work underlined that the optimal onset timing to minimize the metabolic cost of walking is not consistent between studies [1, 3, 21]. However, similar trends were found in different studies for which the optimal onset timing for the reduction of the soleus activation was earlier than the optimal onset timing for the reduction of the metabolic cost of walking [1, 21]. Different studies found that the reduction of the soleus activation is obtained with a bigger positive assistance magnitude, while a medium level of assistance magnitude is more beneficial to reduce the metabolic cost of walking [1, 22]. However, these findings are not compatible with the results obtained in [5, 20, 26]. Some studies tried to define some formulae predicting the performance of the assistance provided by the PAFOs on their users based on the assistive parameters [1, 4]. However, as highlighted in [13], the determination of this formula is not straightforward due to the mutual influence of different parameters and to the fact that the results can be influenced by the different actuation setups and different protocols used in different studies.

In the field of wearable robots, there is a shift towards the use of compliant actuators as actuation principles. Among them, variable stiffness actuators (VSAs) have been shown to be able to minimize large forces due to shocks, to be energy-efficient by storing and releasing energy, and to be robust to external perturbations or unpredictable model errors [27–29]. Despite the well-recognized benefits of VSAs, their employment in wearable robots is still limited due to their design complexity, the increased weight, and the challenging control strategies needed [30]. Among the developed VSAs, the MACCEPA has been already implemented in wearable robots such as lower limbs exoskeletons [31–33] and powered knee [34–36] and ankle orthoses [37–39].

This study evaluates the effects of a MACCEPA-actuated unilateral PAFO, named MAPO (Maccepa Ankle Powered Orthosis) on the walking performance of healthy young users. More specifically, the goal of this study is to evaluate the specific effects of different assistive parameters on the biological effort (i.e., metabolic cost of walking and lower limbs muscle activation) and walking pattern (i.e., lower limbs kinematics and gait parameters) of healthy users. As previously introduced, one of the assistance parameters influencing the PAFO’s assistive performance on the user is the onset timing of the powered push-off. For this reason, the effects of this assistance parameter are investigated with subjects walking with the MAPO. The second parameter investigated in this study is the actuator’s stiffness level. The influence of this parameter on the actuator’s performance has been already shown in several test-bench experiments [37, 40]. In [37] it was shown that no single stiffness level of the ankle actuator used in the MAPO could be defined as optimal based on the actuator’s torque tracking performance, due to the dependence of the benefits of a specific actuator’s stiffness level on both the assistive torque reference and the user’s ankle kinematics. However, the effect of the actuator’s stiffness level on the users has not been yet studied. Thus, a goal of this study is to assess whether the actuator’s stiffness has an influence on the walking performance of healthy users. As previously mentioned, the results of previous studies with bilateral PAFOs [1, 21] showed that the push-off onset time has an influence on the biological effort of healthy users. For this reason, the onset timing of the assistance provided by the MAPO is expected to have an influence on the biological effort of the users. Nevertheless, an additional goal of this study is to evaluate whether similar trends to the ones obtained in [1, 21] can be seen when walking with a unilateral PAFO.

## Methods

### Subjects

Eight healthy male subjects (age 29.8 ±2.6years, height 1.79 ±0.07m, weight 76.5 ±6.5kg, leg length 0.92 ±0.05m) volunteered to participate in the study. All subjects signed the informed consent (in accordance with the General Data Protection Regulation).

### MAPO hardware and control strategy

**Fig. 1:**
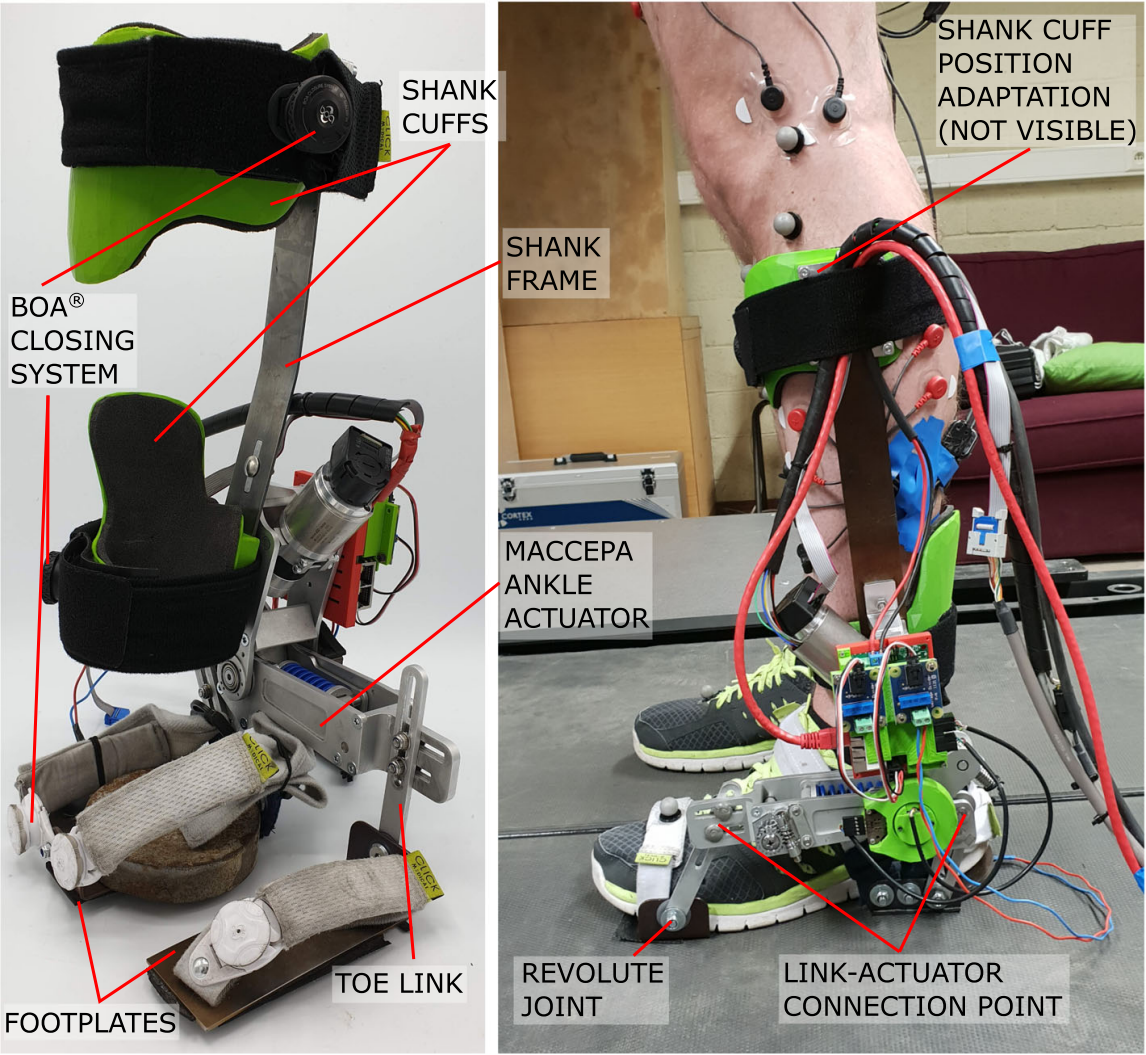
MACCEPA Ankle Powered Orthosis (MAPO). The shank cuffs and the footplates are attached to the user by means of a BOAⓇ closing system. This system allows obtaining a firm connection with a continuous range of adaptability. To provide a more natural walking pattern, the heel plate is slightly bent at the heel, allowing the foot to roll on the ground in the initial phase of the gait cycle. Furthermore, the toe plate is connected to the toe link by means of a revolute joint, which allows the user to rotate about the metatarsophalangeal joint at the end of the stance phase

The PAFO used during the experiments is called MAPO and it is shown in Fig. 1. The design and characterization of the MACCEPA-based ankle actuator implemented in the MAPO have been already presented in detail in [37, 38]. Briefly, the ankle VSA is used to actuate the MAPO in both directions of the sagittal plane (Additional file 1). The actuator’s stiffness level can be changed by modifying the pre-compression (P) of its spring. P is indicated as a percentage of the working length of the spring (spring constant 68.7kN/m, natural length 0.051m, maximal travel of work 0.019m [38]). A level of P equal to 0% indicates that the spring at the actuator’s equilibrium position is uncompressed, while P equal to 100% indicates that the spring is completely compressed at the actuator’s equilibrium position. Thus, the actuator’s behavior is more compliant for lower levels of the spring pre-compression (P). It should be noted that the actuator’s stiffness is not the output stiffness, because the actuator characteristics are not defined as a function of the actuator’s output angle (i.e. the angle between the shank and the foot link), as reported in the Additional file 1 and in [36, 38].

The physical human-robot interface of the MAPO is composed of two 3D printed thermoplastic shank cuffs, a spring steel shank frame, two aluminum foot links, and two spring steel footplates. To be able to adjust the shank interface to different users, the shank cuffs were built in different sizes (to adapt to the user’s calf shape) and the shank frame was built in different lengths (to adapt to the user’s leg length). Furthermore, the position of the higher shank cuff on the shank frame could be changed to increase the adaptability of the MAPO to the user’s shank length (Fig. 1). The placement for the shank cuffs on the user was chosen to increase the stiffness of contact between the user and the MAPO and thus, to improve the torque transmission, as presented in [41]. The adaptability of the footplates to different foot sizes is obtained with the modification of the footplates’ position in the vertical and horizontal directions of the sagittal plane by modifying the attachment point between the foot links and the ankle actuator (Fig. 1) with the mechanism already used in a previous version of the MAPO and presented in details in [39]. The total weight of the MAPO varied between 2.10kg and 2.15kg depending on the size of the shank cuffs and length of the shank frame used for different users.

**Fig. 2:**
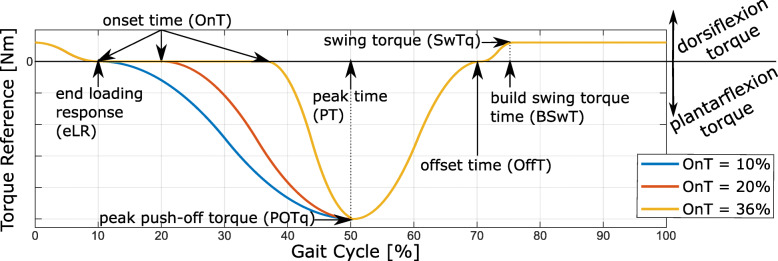
Assistive torque profile provided to the user during a gait cycle. Plantarflexion and dorsiflexion torques are represented by the negative and positive directions, respectively. The assistance parameters that define the torque profile are shown in the figure. The parameters eLR, OnT, PT, OffT, and BSwT are defined as a percentage of a gait cycle. At each new step, the prediction of the gait cycle time was estimated as an average of the gait times measured during 10 steps that preceded the current one to adapt the gait time to possible gait pattern variations during the experiments. The torque profiles corresponding to different OnT are shown in the figure. The torque profiles provided during the third session are equal to the one with OnT set to 20%

During the assisted walking trials, the MAPO assisted the ankle joint of the user in dorsiflexion during the beginning of the stance phase (subphase which is called hereafter the loading response, LR) and swing phases, and in plantarflexion during the push-off phase (Fig. 2). Different assistive profiles were generated by modifying the values of the assistance parameters reported in Fig 2. The structure and implementation of the torque low-level controller of the ankle actuator is reported in details in [37]. The initiation of a new step was detected by means of a force sensing resistor (FSR, SEN-09376 Antratek used with Phidgets Voltage Divider 1121) placed at the heel of the user (Additional file 2).

### Experimental protocol

The protocol consisted of three sessions, each performed on a different day, and separated by at least one day (Table 1). Each session was composed of two parts: some baseline measurements (which were identical in all the sessions) and some treadmill walking trials (which were different in each session, as shown in Table 1). The baseline measurements were performed at the beginning of each session, after an initial warm up. In this part of the sessions, the subjects performed the Maximum Voluntary Isometric Contractions (MVIC) exercises for the soleus (SOL), tibialis anterior (TIB), lateral gastrocnemius (LG), and rectus femoris (REC) in both legs following the guidelines in [42–45]. After this, the *O*_2_ consumption and *CO*_2_ production were recorded while the subject stood still for 5 minutes to calculate the subject’s metabolic cost at rest. At the end of the baseline measurements, retroreflective markers were placed on the user’s lower limbs and a static calibration of the subject standing with straight legs was performed.

**Table 1:** Walking trials performed during the three experimental sessions

**Sess.**	**Trial**	**P**	**eLR**	**OnT**	**PT**	**OffT**	**BSwT**	**POTq**
**1**^***st***^	**NW**	-	-	-	-	-	-	-
	**ZT**							
**2**^***nd***^	**NW2**	-	-	-	-	-	-	-
	**ON10**	40%	10%	10%	50%	70%	75%	0.29 Nm/kg
	**ON20**			20%				
	**ON36**			36%				
**3 **^***rd***^	**NW3**	-	-	-	-	-	-	-
	**PR20**	20%	10%	20%	50%	70%	75%	0.29 Nm/kg
	**PR40**	40%						
	**PR60**	60%						

NW stands for normal walking trial, with number indicating the session; ZT stands for zero torque (i.e., unassisted) walking trial; ON and PR indicate assisted walking trials, with the number indicating the push-off onset time (for ON) and spring pre-compression level (for PR) used. The assistance parameters used during the assisted walking trials are given in the table. The definition of the assistance parameters is shown in Fig. 2. P indicates the spring pre-compression, thus, the stiffness level of the actuator.

The baseline measurements were followed by the treadmill walking trials. For the same subject, the walking speed *v* for these trials was fixed as follows:

$$v = v^{*}\cdot\sqrt{g\cdot l_{0}} $$

where *v*^∗^ is a dimensionless variable, *g* is the gravitational acceleration, and *l*_0_ is the subject’s leg length [46]. The value of *v*^∗^ was set to 0.268, in order to keep the walking speed *v* limited. This choice was taken to ensure the actuator’s capability in tracking the desired torque profile during the walking trials [37]. The walking trials performed in the first session were meant to collect baseline walking data during normal walking (NW) and during the unassisted walking trial called zero torque (ZT). During the ZT walking trial, P was set to 0% to limit unwanted torques being provided by the actuator in the case that a poor tracking of the desired torque profile (i.e. zero torque) would occur (Additional file 1). During the second and third sessions, the subjects performed a normal walking trial (NW2 and NW3, respectively) followed by three assisted walking trials. More specifically, during different assisted walking trials the MAPO provided different assistive profiles (Table 1). All the walking trials lasted 10 minutes, except for NW2 and NW3 which lasted 2 minutes. The goal of the assisted walking trials was to assess the effects of different push-off onset times (OnT, set to 10%, 20%, and 36% of the gait cycle in different walking trials called hereafter ON10, ON20, and ON36, respectively) and the actuator’s stiffness levels (indicated by P and set to 20%, 40%, and 60% in different walking trials called hereafter PR20, PR40, and PR60, respectively). The effect of the two parameters was tested independently to limit the number of walking trials required. The earliest OnT (10%) was selected to start the plantarflexion assistance just after the LR [47], while the latest OnT (36%) was selected based on the results of the study presented in [1]. The effects of these OnT on the actuator’s torque tracking performance was also tested in characterization experiments presented in [37]. The third OnT value (20%) was chosen to assess the effect of a value between the two previously selected. The actuator’s stiffness levels for the third testing session (20%, 40%, and 60%) were selected to be equal to the ones tested in previous characterization experiments on the ankle actuator [37]. In these characterization experiments, it was showed that no single stiffness level can guarantee optimal performance of the actuator. Similarly, in literature, no single OnT demonstrated to minimize both the metabolic cost and muscle activation of the users during walking [13]. For these reasons, the intermediate values of both assistance parameters were chosen as fixed value for the testing session evaluating the effects of the other parameter, as shown in Table 1.

During the walking trials with the MAPO (ZT and assisted), the subjects wore the MAPO only at the left leg. Contrary to the other assistance parameters (Fig. 2 and Table 1), the value of swing torque (SwTq) was self-selected by each subject in a preliminary walking trial. This was done to avoid hurting them by forcing them in an exaggerated dorsiflexion. To determine the suitable amount of torque, the subjects were asked to select a level of assistance which was enough to make them feel that their foot was pushed up during the swing phase, but not too high to make the assistance being uncomfortable or painful. The values selected by the subjects for SwTq varied between 2Nm and 4Nm (mean value ±standard deviation equal to 3.1 ±0.6 Nm). A period of rest was given to the subjects between different walking trials. The walking trials in each session were semi-randomized (to avoid potential bias in the effects of the assisted walking conditions) meaning that NW, NW2, and NW3 were always the first walking trial performed in a session. Furthermore, during NW2 and NW3, only the joint kinematics and gait parameters were recorded.

### Data collection

Twenty-seven retroreflective markers were placed on the subjects’ lower limbs (based on the recommendations in [48, 49]) and their position was recorded using a 10-camera 3D motion-capture system (VICON, Oxford, United Kingdom; 100 Hz sample rate) to estimate the lower limbs position. An instrumented treadmill (X-Mill Dual Belt, ForceLink B.V., Culemborg, The Netherlands; 1000 Hz) measured the ground reaction forces (GRFs), which were synchronized with marker position data. Electromyography (EMG) of the lower limbs muscles was measured by means of surface electrodes (CLEARTRACE EMG electrodes, Conmed, Utica, NY, USA; ME6000 Biomonitor, MEGA Electronics, New Brunswick, NJ, USA; 1000 Hz) placed in a bipolar configuration according to SENIAM guidelines [42]. The *O*_2_ consumption and *CO*_2_ production was measured with a MetaMax 3B breath-by-breath analysis system (Cortex, Germany). During the testing sessions, data were recorded throughout the entire 10 minutes of walking.

### Data analysis

#### Kinematics and gait parameters

The markers trajectories were filtered (4-th order zero-lag Butterworth filter with 6 Hz cut-off frequency) and used to calculate the lower limb joint angles in the three planes of rotation using a Cardan XYZ decomposition of the rotation matrices based on right-handed local orthonormal reference frames for the lower limbs segments [48–50]. The vertical GRFs were used to segment the joint trajectories into gait cycles by means of the identification of the heel strike (HS) and toe-off (TO) at each leg. The segmented joint trajectories were time-normalized to the gait cycle (0-100%) by means of linear interpolation and divided into 10 groups each containing the data recorded during 1 minute of walking. The trajectories belonging to each group were averaged to obtain a mean joint trajectory for each minute of walking. Incorrect standing poses of the subjects during the static calibration can influence the computation of the joint kinematics. Thus, the static calibration joint angles and the static calibration poses were assessed to determine whether the subject was standing in a correct position. Incorrect standing positions were defined as poses in which the subjects did not stand with both legs straight. An example of an incorrect standing position is the one of subjects who stood with a knee flexed. If these incorrect poses were observed, the joint kinematics of the related walking trials were inspected to check whether an offset could be noted between the left and right kinematics. If an offset was found also in these data, it was assumed that the difference in the angles at the standing pose was due to an incorrect pose which needed to be corrected. The correction was performed by calculating the difference between the angles of the joint at the two legs and by using it to correct the offset at the joint angles obtained from the walking trials. In addition to this, the joint kinematics of all the walking trials were assessed in terms of joint ROM (i.e., maximum minus minimum measured value) to reduce the effects that small differences in the markers placement on the lower limbs could have on the results. The joint ROMs obtained at the second minute in the normal walking trial of a session were subtracted from the ones calculated at different minutes of the walking trials. The variables calculated with this procedure are called hereafter *Δ*ROM. *Δ*ROM were used in the analysis to avoid a possible influence of an inconsistent placement of the retroreflective markers on the calculation of the joint ROM in different sessions. The joint *Δ*ROM analyzed in different walking trials were the sagittal hip and knee ROM during the entire stride, and the ROM of the sagittal ankle joint during stance and swing. In addition to these parameters, the sagittal ankle heel strike ROM (HS ROM) was computed as the difference between the ankle angle at HS and the maximum plantarflexion angle measured in the first 10% of the gait cycle. Similarly to the joint trajectories, the measured gait spatio-temporal parameters were divided into 10 groups containing the data of one minute and the average of each group was calculated. The stride length was calculated by multiplying the time between two consecutive HS of the same leg by the speed of the treadmill. The stride width was calculated as the largest medio-lateral distance between the markers placed at the calcaneus during the strides. The stance percentage was defined as the percentage of the gait cycle which was spent in stance.

#### Electromyography

Based on the recommendations reported in [43, 51], the raw EMG data were filtered (bandpass digital non-causal FIR linear phase filter, stop-band frequencies 10 and 500 Hz, pass-band frequencies 15 and 400 Hz, stop-band attenuation 80 dB and 1 ms sampling time), full wave rectified, and smoothened by means of a moving average algorithm (window width of 200 ms and of 500 ms for walking and MVIC trials, respectively). The factors for the normalization of each muscle were calculated as the mean amplitude of the highest portion of the processed MVIC signals and not as a single peak data point [43]. The processed EMG signals from the walking trials were normalized based on these factors. The normalized EMG signals were manually synchronized with the kinematics data by making the right TO coincide with the end of the activation of the right LG. The EMG signals were then segmented into gait cycles, time-normalized between 0 and 100% of the gait cycle, divided into 10 groups containing 1 minute of data, and averaged to obtain an average muscle activity per minute. The root-mean-square (RMS) amplitude of the SOL and LG was calculated in the stance phase. The RMS amplitude of the REC was calculated in both the stance and the swing phase. The RMS amplitude of the TIB was calculated in the LR phase (from 0% to 10% of the gait cycle) and in the swing phase.

It should be highlighted that the use of MVIC exercises for the normalization is based on the hypothesis that a subject will produce the same amount of force during the same MVIC exercises on different days. However, for untrained subjects it could be difficult to produce a true MVIC contraction [43]. In other studies, the normalization of the EMG data was based on the maximum amplitude of the EMG signal measured during a reference walking condition [1, 5, 21]. It is important to point out that these two normalization methods will result in different outcomes if the subjects do not use the same percentage of muscle force during the reference walking trial as compared to the maximal force measured during the MVIC exercises.

#### Metabolic cost

The metabolic cost of walking was estimated from the *O*_2_ consumption and *CO*_2_ production by means of the simplified Brockway formula [18, 19, 52], divided into 10 groups containing data of 1 minute of walking trial, and averaged to find, for each minute, a mean gross metabolic cost. The net metabolic cost of walking was computed by subtracting the 5-minute rest metabolic cost from the gross metabolic cost of walking and dividing the result by the subject’s weight.

### Statistical analysis

The statistical analysis was performed in the SASⓇ University Edition software [53]. A linear mixed model with repeated measures on the conditions factor and a random intercept effect to account for correlations within subjects was used for all dependent variables. This model was chosen over conventional repeated-measures ANOVA because some data were missing due to issues with the equipment, and mixed models can make use of all available data and are unbiased under the missing-ad-random assumption [54]. The confidence levels for all the statistical analyses were set to 95%, which lead to a significance level *α* = 0.05. During each analysis, the assumption of residual normality was visually inspected and, if violated, data were transformed using monotone functions. The effects of different walking trials were compared among each other at minutes 1, 4, 7, and 10 of the walking trials. When a statistical difference was found between the analyzed conditions, post-hoc comparisons were done by using Tukey test. Regarding the analysis of the data that were corrected for normality, the *p*-value of the transformed data is reported. In some cases, none of the applied data transformations properly corrected the normality of the data. However, in these cases, the statistical results obtained under different transformations were consistent. Thus, in these cases, the result of the analysis from the original data was not affected by the fact that the normality assumption was violated. For this reason, the *p*-values of the original data are reported.

## Results

### Metabolic cost

**Fig. 3:**
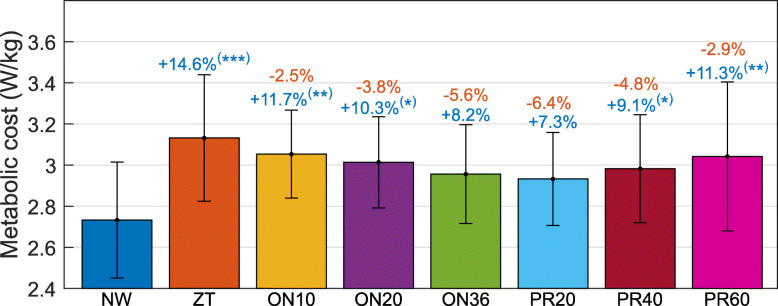
Comparison of the net metabolic cost of walking in different walking conditions at minute 10. The amount of increase and decrease in the net metabolic costs as compared to NW and ZT is reported (blue for NW and orange for ZT). Statistically significant difference in the net metabolic cost of the walking trials as compared to NW and ZT is reported with blue and orange asterisks, respectively. * indicates p ≤0.05, ** indicates p ≤0.01, *** indicates p ≤0.001. It should be noted that the graph does not start from zero

During the first minute of walking, the average net metabolic costs of the walking trials with the MAPO were from 10.3% to 14.3% higher than the one obtained during NW (Table 2 and Additional file 3). These increases in the net metabolic costs were statistically significant in ON10 (p=0.03), ON20 (p=0.04), and PR20 (p=0.01). Furthermore, the net metabolic costs of the assisted walking trials at the first minute were similar (from 0.1% lower to 3.5% higher, p >0.98) to the one during ZT. Contrary to this, at minute 10, the net metabolic costs of the assisted walking trials were from 7.3% to 11.7% higher (p >0.10 for ON36 and PR20, p ≤0.05 for the other assisted walking trials) than the one obtained during NW, but they were from 2.5% to 6.4% lower (p >0.34) than the one obtained during ZT, as shown in Fig. 3. No statistically significant differences were found between the net metabolic costs of assisted trials during the last minute of walking (p >0.91). It should be noted that these data capture a delayed metabolic response. For this reason, although both data from the first and the last minute of walking are provided, the results for the last minute of walking are more informative for measuring the specific effect of the MAPO on the users.

**Table 2:** Experimental data collected at the first and last minute of each walking trial

**Trial**	**Min.**	**Net met. cost**	**RMS SOL**	**Ankle HS**	**Ankle swing**	Hip	**Hip**	**Stance perc**	**Stride length**	**Stride length**
		**[W/kg]**	**stance LEFT**	***Δ*ROM LEFT**	***Δ*ROM LEFT**	*Δ*ROM LEFT	***Δ*ROM RIGHT**	**LEFT [%]**	**LEFT [m]**	**RIGHT [m]**
			**[%MVIC]**	**[deg]**	**[deg]**	[deg]	**[deg]**			
**NW**	**1**	2.63 ±0.29	0.42 ±0.15	-0.03 ±0.13	-0.57 ±1.18	-0.83 ±0.50	-0.60 ±1.10	64.94 ±1.29	1.08 ±0.11	1.08 ±0.11
	**10**	2.73 ±0.28	0.40 ±0.15	0.13 ±0.39	-0.18 ±0.61	0.14 ±1.28	0.08 ±1.13	64.46 ±1.06	1.10 ±0.12	1.10 ±0.12
**ZT**	**1**	2.90 ±0.45	0.43 ±0.17	-0.33 ±1.37	-2.45 ±1.46	2.26 ±2.69	0.37 ±1.95	62.83 ±1.88	1.11 ±0.13	1.11 ±0.13
	**10**	3.13 ±0.31	0.39 ±0.11	-1.05 ±1.77	-2.85 ±1.47	0.97 ±1.98	0.02 ±1.58	62.89 ±1.20	1.10 ±0.14	1.10 ±0.14
**ON10**	**1**	2.98 ±0.27	0.28 ±0.09	2.12 ±1.73	4.07 ±4.89	4.54 ±2.91	1.16 ±1.67	62.96 ±1.48	1.15 ±0.10	1.15 ±0.10
	**10**	3.05 ±0.21	0.27 ±0.06	1.83 ±1.49	1.63 ±3.79	5.26 ±2.53	1.73 ±1.67	62.51 ±1.15	1.16 ±0.11	1.16 ±0.11
**ON20**	**1**	2.96 ±0.23	0.30 ±0.12	2.24 ±1.86	2.57 ±3.66	4.31 ±1.67	1.37 ±1.87	62.79 ±2.24	1.15 ±0.10	1.15 ±0.10
	**10**	3.01 ±0.22	0.28 ±0.10	1.65 ±2.05	1.48 ±3.85	5.42 ±2.10	1.73 ±2.08	62.66 ±1.46	1.15 ±0.11	1.15 ±0.11
**ON36**	**1**	2.89 ±0.24	0.31 ±0.13	1.99 ±1.86	2.79 ±3.05	3.78 ±2.36	1.70 ±1.93	63.22 ±1.34	1.14 ±0.10	1.14 ±0.10
	**10**	2.96 ±0.24	0.29 ±0.11	1.69 ±1.43	0.93 ±3.20	4.91 ±3.21	2.29 ±2.26	62.98 ±1.35	1.15 ±0.10	1.15 ±0.11
**PR20**	**1**	3.00 ±0.33	0.30 ±0.08	1.25 ±1.00	2.05 ±2.41	2.34 ±3.25	0.60 ±1.88	62.96 ±1.19	1.10 ±0.11	1.10 ±0.11
	**10**	2.93 ±0.23	0.27 ±0.06	1.53 ±1.05	1.41 ±2.55	4.51 ±2.71	2.58 ±2.58	63.14 ±1.28	1.15 ±0.13	1.15 ±0.12
**PR40**	**1**	2.93 ±0.32	0.28 ±0.07	1.02 ±1.09	1.64 ±2.96	3.63 ±2.55	1.66 ±1.68	62.77 ±1.36	1.12 ±0.11	1.12 ±0.11
	**10**	2.98 ±0.26	0.28 ±0.07	1.06 ±0.89	1.86 ±3.36	4.14 ±3.00	2.06 ±2.95	62.96 ±0.88	1.14 ±0.11	1.14 ±0.11
**PR60**	**1**	2.91 ±0.27	0.33 ±0.16	1.90 ±1.66	1.97 ±3.70	2.89 ±2.78	1.44 ±2.45	62.43 ±1.34	1.14 ±0.10	1.13 ±0.10
	**10**	3.04 ±0.36	0.29 ±0.09	1.86 ±1.58	0.32 ±3.83	4.18 ±2.96	2.04 ±2.98	62.40 ±0.99	1.15 ±0.10	1.15 ±0.10

Data are given as mean ±standard deviation. The RMS of the left SOL is normalized such that 1 corresponds to the maximum activation recorded during the MVIC exercises. The ankle HS and swing *Δ*ROM and the hip *Δ*ROM are calculated by subtracting the ROM measured during the normal walking trial at minute 2 from the ROM measured during the considered walking trial at minute 1 or 10, respectively.

### Electromyography

**Fig. 4:**
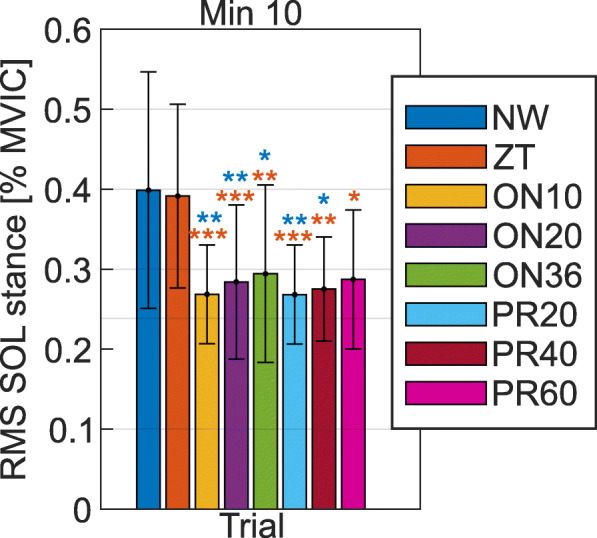
RMS activity of the left SOL during stance in different walking conditions. Data are given for the last minute of walking and are normalized such that 1 corresponds to the maximum activation recorded during the MVIC exercises. Statistically significant difference in the muscle activity as compared to NW and ZT is reported with blue and orange asterisks, respectively. * indicates p ≤0.05, ** indicates p ≤0.01, and *** indicates p ≤0.001

During ZT, the stance RMS activity of the left SOL was similar to the one in NW at all the assessed minutes (p >0.97). Contrary to this, the RMS activity of the left SOL during stance was reduced in the assisted walking trials as compared to NW and ZT, at all the assessed minutes. At the last minute of walking, the reductions, as compared to NW, were equal to 32.6% in ON10, 28.7% in ON20, 26.2% in ON36, 32.7% in PR20, 31.0% in PR40, and 28.0% in PR60 (Fig. 4 and Table 2). The reductions in the stance RMS of the left SOL as compared to NW at the last minute of walking were statistically significant in all the assisted walking trials (p ≤0.02) except for PR60 (p=0.12). However, the stance RMS of the left SOL in any of the assisted walking trials was statistically significantly lower than the one in ZT (p ≤0.01). No statistically significant difference was found between the left SOL RMS activity during stance between different assisted walking trials (p >0.52). Walking with the MAPO did not have any statistically significant effect on the TIB, LG, and REC of both legs, and on the right SOL across different walking trials (p >0.06, see Additional file 4 and Additional file 5). The only exception was the RMS activity of the left REC during swing in ON10, which was significantly lower than the one in NW at minute 1 (p=0.04).

### Joint kinematics

**Fig. 5:**
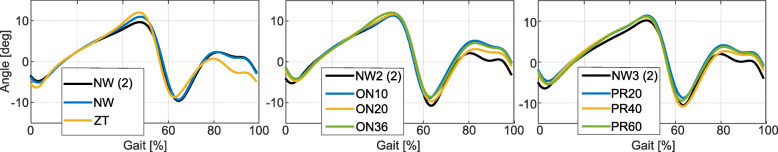
Left ankle joints kinematics during different walking conditions. Every column represents a different session. The joint trajectories are given for the last minute of walking. For each session, the joints trajectories for the second minute of walking in the NW condition are also given (NW(2), NW2(2), and NW3(2)). As compared to NW, the subjects showed an increased HS *Δ*ROM and swing *Δ*ROM in the assisted walking trials and a decreased HS *Δ* ROM and swing *Δ*ROM in ZT

The left ankle HS *Δ*ROM was decreased at all the assessed minutes in ZT as compared to NW (Fig. 5 and Additional file 8), but the decreases were not statistically significant (p >0.56). Contrary to this, the left ankle HS *Δ*ROM was increased in all the assisted walking trials as compared to NW and ZT. At the last minute of walking (Table 2 and Additional file 8), the increases obtained in the assisted walking trials were not statistically significantly different from the ones in NW (p >0.23), while they were statistically significant as compared to ZT in all the assisted walking trials (p ≤0.02), except for PR40 (p=0.08). No statistically significant difference was found in the left ankle HS *Δ*ROM between assisted walking trials for the assessed minutes (p >0.75).

From the first to the last minute of walking in ZT, the left ankle swing *Δ*ROM decreased as compared to the one in NW (Additional file 8 and Table 2). However, the differences between the two walking trials were not statistically significant (p >0.30). Contrary to this, the left ankle swing *Δ*ROM was increased in the assisted walking trials at all the assessed minutes as compared to NW and ZT. At the first minute of walking, the increase was statistically significant only in ON10 as compared to NW (p=0.03, for all the other assisted walking trials p >0.43), while it was statistically significant in all the assisted walking trials as compared to ZT (p ≤0.05). A different scenario was obtained at the last minute of walking. At this minute, although the left ankle swing *Δ*ROM was, on average, still higher in the assisted walking trials than in NW and ZT (Table 2, Fig. 5, and Additional file 8), this increase was statistically significant only in ON10 as compared to ZT (p=0.02; other assisted conditions as compared to ZT have p >0.08; all assisted conditions as compared to NW have p >0.69).

Some effects of walking with the MAPO could also be noticed at the hip joint of the users. The left hip *Δ*ROM was increased in all the walking trials with the MAPO (ZT and assisted) as compared to the one during NW at all the assessed minutes. At the last minute of walking, the left hip *Δ*ROM was slightly higher in ZT as compared to NW, but the difference was not statistically significant (p=0.99). Contrary to this, the left hip *Δ*ROM at the last minute of walking was higher in all the assisted walking trials as compared to the one measured in NW (Table 2), with the increase being statistically significant in all the assisted walking trials (p ≤0.01). As compared to ZT, the increase in the left hip *Δ*ROM was statistically significant in ON10, ON20, ON36, and PR20 (p ≤0.04), but not in PR40 and PR60 (p >0.05). No statistically significant changes were found between the values of the left hip *Δ*ROM in different assisted walking trials at the assessed minutes (p >0.16). Contrary to the left hip *Δ*ROM, the increase in the right hip *Δ*ROM in the assisted walking trials as compared to NW was not statistically significant in any of the assessed minutes (p >0.10). No statistically significant difference was found between the right hip *Δ*ROM in NW and ZT (p >0.05) and between different assisted walking trials (p >0.05) at any of the assessed minutes (Table 2).

Across different walking conditions, no noticeable effects of walking with the MAPO could be found in the right ankle HS *Δ*ROM and swing *Δ*ROM, and in the left and right knee *Δ*ROM and ankle stance *Δ*ROM (p >0.08, see Additional file 6 and Additional file 7).

### Gait parameters

**Fig. 6:**
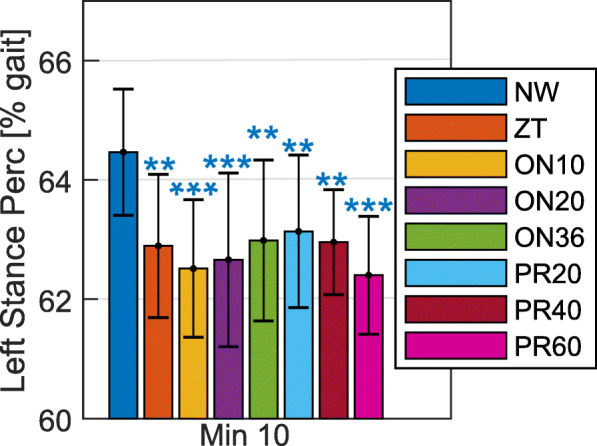
Left stance percentage during different walking conditions. The average left stance percentage is reported for the last minute of the walking trials. Statistically significant differences, as compared to NW, are reported with ** to indicate p ≤0.01, and *** to indicate p ≤0.001. It should be noted that the graph does not start from zero

As compared to NW, when walking with the MAPO, the subjects reduced the left stance percentage at all the assessed minutes (Table 2). All the reductions were statistically significant (p ≤0.04) except for the reduction obtained at minute 1 and minute 7 during ON36 (p >0.07). The reductions at the last minute of walking are shown in Fig. 6. No statistically significant difference was found in the left stance percentage in any of the assisted walking trials as compared to ZT (p >0.79) or other assisted walking trials (p >0.50).

The subjects tended to increase the left and right stride length during the assisted walking trials as compared to NW at all the assessed minutes (Table 2 and Additional file 9). However, the increase was statistically significant only in ON10 at minute 1 and minute 4 for both the left and right leg (p ≤0.04) and in ON20 at minute 1 at the left leg (p=0.04), but not for the other conditions (p >0.05). No statistically significant difference was found between the stride length in ZT and NW in both the left and right leg (p >0.05).

No significant effects were found in the stride width and the right stance percentage across different walking trials at the assessed minutes (stride width: p >0.79, right stance percentage: p >0.10, see Additional file 9).

## Discussion

The results presented in the previous section show that the MAPO tended to be more metabolically efficient for all the assisted walking trials as compared to ZT (Table 2 and Fig. 3). This suggests that the assistance provided by the MAPO can compensate for the added weight of the device, which, being located distally on the body, caused an increase in the metabolic cost as compared to NW (Table 2, Fig. 3, and Additional file 3).

There seems to be a trend for later push-off onset timings and more compliant actuator’s settings (between the conditions tested) being more favorable in terms of reducing the net metabolic cost. This is highlighted also by the fact that the increase in the net metabolic cost of walking in the assisted trials gets smaller with time in all the assisted walking trials (as compared to NW) but the stiffer configuration of the actuator (PR60), for which this increase is bigger at minute 10 than at minute 1.

Table 2 and Fig. 4 show the trend of reduced activation of the assisted SOL during stance with earlier push-off onset timings and more compliant actuator’s settings, among the ones that were tested.

The trend towards a bigger reduction in the net metabolic cost of walking with the latest push-off onset time (among the ones tested in this study) and a bigger reduction in the SOL activation with earlier push-off onset timings is consistent with the outcomes reported in other studies with healthy subjects [1, 3, 13, 21]. The difference between these studies and the work presented in this article lies in the PAFO configuration (while the MAPO is a unilateral PAFO, bilateral PAFOs were used in [1, 3, 21]) and the walking speed tested (the subjects participating in the experiments reported in [1, 3, 21] walked at higher speeds than the ones at which the MAPOwas tested). Because of these differences in the experimental protocols, the results obtained with the MAPO also suggest that the effects of the push-off onset time are maintained at different walking speeds and for both configurations of the PAFOs.

The results obtained suggest that the subjects did not reach a steady-state during the 10 minutes of walking, which is coherent with the adaptation times obtained in other works [13]. For this reason, it is possible that larger reductions (as compared to ZT) in the net metabolic cost of the assisted walking conditions would have been found if the subjects had walked for a longer time in each condition. Furthermore, the peak push-off torque provided during the experiments was lower than the one provided during similar studies by other research groups [5, 16, 20, 55–57]. Providing higher peak torque could lead to higher reductions in the metabolic cost of walking and soleus muscle activation.

A recent study [58] showed that the uni-/bi-lateral configuration of the actuator influences the performance of the actuator in reducing the metabolic cost of walking in healthy subjects. More specifically, the study showed that a given amount of assistance from a PAFO is more efficient in reducing the metabolic cost of walking when it is evenly distributed over both legs (i.e. the total assistance is provided in a bilateral configuration) as compared to being provided unilaterally (i.e. the total assistance is provided unilaterally with the users wearing the PAFOs on both legs). Thus, based on the results found in [58], bigger reductions of the metabolic cost during the assisted walking trials (as compared to ZT) might have been obtained if the same total assistance would have been distributed over the two legs.

While the plantarflexion assistance provided at the push-off resulted in a reduction of the SOL during the assisted walking trials, the assistance provided in dorsiflexion during the swing and LR phases did not reduce the activity of the left TIB in any of the assisted phases (Additional file 4 and Additional file 5). This result is in contrast with what was found in [59], where the dorsiflexion assistance provided to healthy subjects reduced the TIB activity during LR, but not during swing. In [59], these different effects were explained by the reasoning that there is no penalty in having exaggerated ankle dorsiflexion in swing (thus, there is no benefit in reducing the TIB activation in this phase), while exaggerated ankle dorsiflexion during the LR phase could cause instability. The increase in the ankle dorsiflexion obtained with the MAPO in these two phases was lower than the one obtained in [59]. This could be due to the relatively low peak torque provided in dorsiflexion. Thus, the increase in the ankle dorsiflexion due to the low powered assistance provided by the MAPO during LR did not cause instability in the subjects, who did not reduce the activity of the TIB in this phase.

In some studies, after a period of adaptation, the powered assistance provided at the push-off did not have any effect on the activity of more proximal muscles [14, 15, 17, 59]. As opposed to this, other studies found that the assistance provided at the push-off phase was reducing the activity of some muscles at the hip joint [2, 11, 22, 23]. In this study, no significant effects were found at the left REC at the end of the walking trials (Additional file 4 and Additional file 5). Thus, the results of this study belong to the first group of studies above mentioned, in which the effects of the assistance provided at the ankle joint during push-off resulted in a partial replacement of plantarflexor muscular effort, rather than in their augmentation in favor of more proximal muscles.

The reduction of the stance percentage in the left leg in the assisted walking trials (Table 2 and Fig. 6) could be due to the push-off assistance, which could have forced the subjects to move up the beginning of the swing phase. However, this effect could also be an indicator that the subjects did not have enough time to familiarize themselves with the powered assistance and, thus, they tended to reduce the amount of time in which the left leg supports the bodyweight. This is highlighted by the fact the trend for a bigger reduction of the stance percentage for earlier push-off onset timings and stiffer configurations of the actuator between the ones tested (Table 2 and Fig. 6). These are the conditions that lead to a smaller reduction in the metabolic cost of walking. The fact that the stance percentage is reduced also in ZT could be because this trial was the first walking trial in which the subjects wore the MAPO and could familiarize with the extra weight and the kinematic constraints that walking with a device with a single degree of freedom imposes on the user.

During the assisted walking trials, the subjects increased the hip *Δ*ROM in both legs (Additional file 7). However, this increase came from a bigger hip flexion in the first part of the stance and swing at the left leg and from an increased maximum hip extension during stance at the right leg. This different effect could be related to the fact that the subjects took slightly longer steps while walking with the assistive MAPO, but they tended to show a shorter stance phase in the left leg during the assisted walking trials (Table 2).

Some limitations of the study performed in this work should be highlighted. First of all, the experiments were performed only on eight subjects. Although this number of subjects is comparable with the one of other similar studies [2, 6, 7, 20, 22, 25, 57, 60], it leads to a low statistical power of the hypothesis test. This number of subjects was chosen as a trade-off between the reliability of the results obtained in this study and the time needed for the data collection and analysis. Nevertheless, the trends found in the presented study can serve as input for follow-up experiments with a higher number of healthy participants and aiming to clarify the effects of assistance parameters on the walking performance.

During the experiments, the EMG measurement device could not be synchronized with the other measurement devices. For this reason, as previously reported, in the post-analysis of the EMG data, we had to synchronize manually these sets of data. This manual synchronization method might have introduced some errors in the data analysis, thus, it is a limitation of this study. However, the possible synchronization error would have affected all the data at the same level, thus it would have not influenced the results obtained regarding the effects of different walking conditions.

Another limitation of the experiments performed in this study is the unreliability of using an FSR to detect the gait cycle [61]. This method was implemented in the experimental protocol due to the simplicity of the detection algorithm needed. In future work, a different gait detection algorithm, as the ones presented in [62–64], could be used to improve the performance and the robustness of the actuator’s controller.

For the sake of simplicity in the development of the actuator’s controller, only the push-off onset timing (within a limited range of values) was controlled in the experiments performed in this work. In future works, later push-off onset timings should be tested for slower walking speeds and the resulting effects should be compared with the ones obtained at higher walking speeds. Furthermore, it is known that many parameters mutually influence the effects of powered walking on the user [13]. For this reason, the influence of other assistance parameters (for example, the average power provided by the MAPO during one stride) on the users should be evaluated in future work. In addition to this, the relationship between the actuator’s parameters (for example its stiffness or the spring characteristics) and the user’s parameters (for example his/her weight or height) could be investigated in future work.

## Conclusions

This study evaluated the effects of different onset timings of the push-off torque and different actuator’s stiffness levels on healthy young users during walking with a unilateral PAFO, more specifically the MAPO (Maccepa Ankle Powered Orthosis).

The results obtained for the push-off onset timing show similar trends in the effects on the metabolic cost of walking and the soleus muscle activation as the ones found in other studies for a bilateral configuration of the PAFO and higher walking speeds. Thus, a conclusion of this study is that similar trends seem to be maintained for the effects of the push-off onset time for different PAFO configurations and different walking speeds.

Furthermore, this study is a first attempt to analyze the influence of the actuator’s stiffness level on the assistance provided to the user. The results obtained show that, among the levels tested, a more compliant configuration of the actuator seems to be more beneficial in terms of the reduction of the metabolic cost of walking and soleus muscle activation during powered walking.

In addition, it is showed in this study that a bigger reduction of the stance percentage in the leg which was wearing the MAPO was obtained with stiffer actuator configurations and earlier push-off onset timings (among the ones tested). Being these conditions the ones resulting in lower reductions of the metabolic cost of walking, this result might be an indication that the subjects need more time to adapt to the powered assistance in these configurations.

## Supplementary information

**Additional file 1** Document 1. Additional information on MACCEPA

**Additional file 2** Document 2. Algorithm used for heel strike detection.

**Additional file 3** Figure S1. Net metabolic cost of walking in different walking conditions at the first and last minute of walking. To improve the readability of the graph, the values of the net metabolic costs of normal walking (NW) and zero torque (ZT) are reported across the other conditions with a horizontal dashed line (blue for NW and orange for ZT). Statistically significant difference in the net metabolic cost of the walking trials as compared to NW and ZT is reported with blue and orange asterisks, respectively. * indicates p ≤0.05, ** indicates p ≤0.01, *** indicates p ≤0.001.

**Additional file 4** Figure S2. Envelopes of the soleus (SOL), tibialis anterior (TIB), lateral gastrocnemius (LG), and rectus femoris (REC) activity at the leg wearing the MAPO during different walking conditions at the last minute of walking. The envelopes are normalized such that 1 corresponds to the maximum value obtained during the MVIC exercises and they are the averaged envelopes of the different subjects.

**Additional file 5** Table S1. RMS of the activity of the lower limbs muscles across walking conditions at the last minute of walking.

**Additional file 6** Figure S3. Hip, knee, and ankle joints kinematics in the sagittal plane at the assisted leg during different walking conditions. The joint trajectories are given for the last minute of walking. For each session (each row), the joints trajectories for the second minute of walking in the NW condition is also given (NW(2), NW2(2), and NW3(2)).

**Additional file 7** Table S2. Kinematics parameters measured in different walking conditions at the last minute of walking.

**Additional file 8** Figure S4. Left ankle HS ROM and left ankle swing ROM during different walking conditions at the first and last minute of walking. Statistically significant differences as compared to NW and ZT are reported with blue and orange asterisks, respectively. * indicates p ≤0.05, ** indicates p ≤0.01, and *** indicates p ≤0.001.

**Additional file 9** Table S3. Gait parameters measured in different walking conditions at the last minute of walking.

## Data Availability

Data generated or analyzed during this study which are not included in this published article and its supplementary information files are available from the corresponding author on reasonable request.

## Abbreviations

PAFO: Powered ankle-foot orthosis
MAPO: Maccepa ankle powered orthosis
VSA: Variable stiffness actuator
MACCEPA: Mechanically adjustable compliance and controllable equilibrium position actuator
MVIC: Maximum voluntary isometric contractions
SOL: Soleus
TIB: Tibialis anterior
LG: Lateral gastrocnemius
REC: Rectus femoris
OnT: Onset time of the push-off torque
P: Spring pre-compression level
NW: Normal walking
ZT: Zero torque walking
ON10, ON20, ON36: Assisted walking trials with OnT set to 10%, 20%, and 36% of the gait cycle, respectively
PR20, PR40, PR60: Assisted walking trials with P set to 20%, 40%, and 60%, respectively
SwTq: Swing torque
GRF: Ground reaction force
EMG: Electromyography
ROM: Range of motion
HS: Heel strike
TO: Toe-off
RMS: Root-mean-square
LR: Loading response
FSR: Force sensing resistor
