# Supplementary material for: Walking with a powered ankle-foot orthosis: the effects of actuation timing and stiffness level on healthy users
Source: J Neuroeng Rehabil. 2020 Jul 17;17:98. doi: 10.1186/s12984-020-00723-0 (PMC7367242; doi:10.1186/s12984-020-00723-0)
Supplement: Supplementary file 1 — Additional file 1 Document 1. Additional information on MACCEPA [file 12984_2020_723_MOESM1_ESM.pdf]

MACCEPA additional information

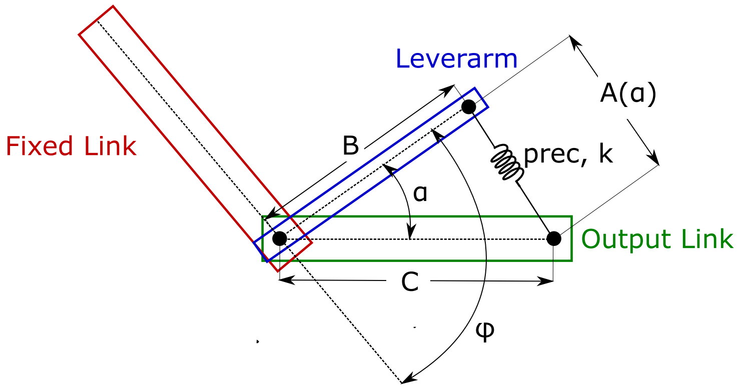

Figure 1: Schematic drawing of the MACCEPA and its parameters [1]. B and C are the distances from the joint axis of the attachment points of the MACCEPA spring on the leverarm and the output link, respectively; k and prec are the MACCEPA spring constant and pre-compression, respectively; α is called the deflection angle; φ is the equilibrium position angle, also called leverarm angle. $A(\alpha)$ is calculated as shown in Eq. 2.

The MACCEPA is a torque-controlled, variable stiffness actuator (VSA) whose working principle is described in details in [1]. The schematic drawing of the MACCEPA and the MACCEPA's parameters are shown in Fig. 1. As shown in Fig. 1, the MACCEPA consists of three bodies (*fixed link*, *output link*, and *leverarm*) pivoting around a common axis. A spring is attached between the leverarm and the output link. The torque (τ) provided by the MACCEPA is defined by its parameters as shown in Eq. 1:

$$\tau = f_{\mathrm{MACC}}(\alpha, \mathrm{prec}) = k \cdot B \cdot C \cdot \sin(\alpha) \cdot \left(1 + \frac{\mathrm{prec} - |C - B|}{A(\alpha)}\right) \tag{1}$$

where $A(\alpha)$ is calculated as follows:

$$A(\alpha) = \sqrt{B^2 + C^2 - 2 \cdot B \cdot C \cdot \cos(\alpha)} \tag{2}$$

In the MACCEPA, the configuration in which the leverarm and the output link of the actuator are aligned, thus, in which the deflection angle (α) is equal to zero, is called the equilibrium position. In this configuration, the force created in the MACCEPA spring by the spring pre-compression (prec) generates no torque in the actuator, due to the alignment of the leverarm and output link.

Figure 2 shows the MACCEPA-based actuator used in the experiments presented in the article. More details on the actuator's design can be found in [2].

The spring pre-compression (prec) in Eq. 1 is measured in meters. However, the level of spring pre-compression (P) is defined as a percentage of the working length of the MACCEPA spring. Specifically, the level of spring pre-compression is defined to be equal to 0% when the length of the spring at the actuator's equilibrium position is equal to the length of the uncompressed spring (i.e. the natural length of the spring). Similarly, the level of spring pre-compression (P) is equal to 100%

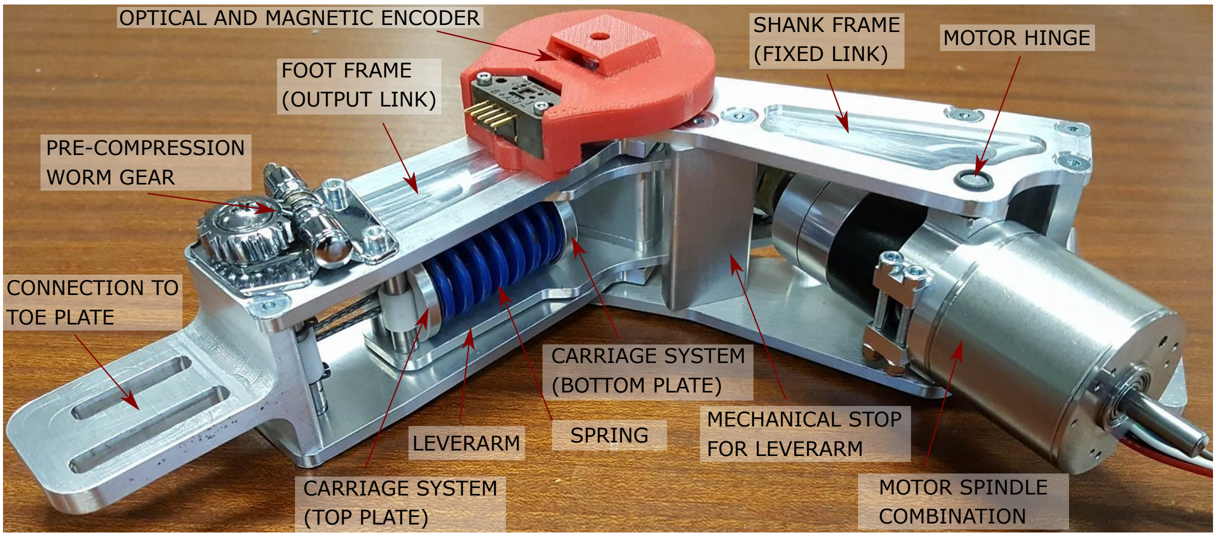

Figure 2: The MACCEPA-based ankle actuator. The different components of the actuator are shown in the figure.

when the length of the spring at the equilibrium position is equal to the length of the spring when it is completely compressed.

The pre-compression of the MACCEPA spring modifies the behavior of the MACCEPA. Higher spring pre-compression levels result in stiffer behavior of the actuator. On the contrary, the actuator is more compliant with lower spring pre-compression levels. In other words, the MACCEPA spring pre-compression determines the amount of torque exerted by the MACCEPA (with fixed values for the parameters B, C and k) for a fixed value of the deflection angle. Figure 3 shows the effect of the spring pre-compression level (P) on the behavior of the MACCEPA-based actuator (Fig. 2), i.e. its deflection angle-torque characteristics.

As it can be seen from Fig. 3, for each pre-compression level, both the MACCEPA output torque (τ) and apparent stiffness are non-linear functions of the deflection angle (α) [3]. The deflection angle-torque characteristics can be approximated with a third order polynomial, thus, the deflection angle-stiffness characteristics can be approximated using a second order polynomial as presented in [3]. The resulting approximated deflection angle-stiffness characteristics of the MACCEPA-based ankle actuator for the spring pre-compression levels used during the experiments (20%, 40%, and 60%) are shown in Fig. 4.

References

[1] R. Van Ham, B. Vanderborght, M. Van Damme, B. Verrelst, and D. Lefeber, "MACCEPA, the mechanically adjustable compliance and controllable equilibrium position actuator: Design and implementation in a biped robot," *Robotics and Autonomous Systems*, vol. 55, no. 10, pp. 761–768, 2007.

[2] M. Moltedo, T. Bacek, K. Langlois, K. Junius, B. Vanderborght, and D. Lefeber, "Design and experimental evaluation of a lightweight, high-torque and compliant actuator for an active ankle foot orthosis," in *2017 International Conference on Rehabilitation Robotics (ICORR)*. London, UK: IEEE, jul 2017, pp. 283–288.

[3] T. Bacek, M. Moltedo, C. Rodriguez-Guerrero, J. Geeroms, B. Vanderborght, and D. Lefeber, "Design and Evaluation of a Torque-Controllable Knee Joint Actuator with Adjustable Series Compliance and Parallel Elasticity," *Mechanism and Machine Theory*, vol. 130, pp. 71–85, 2018.

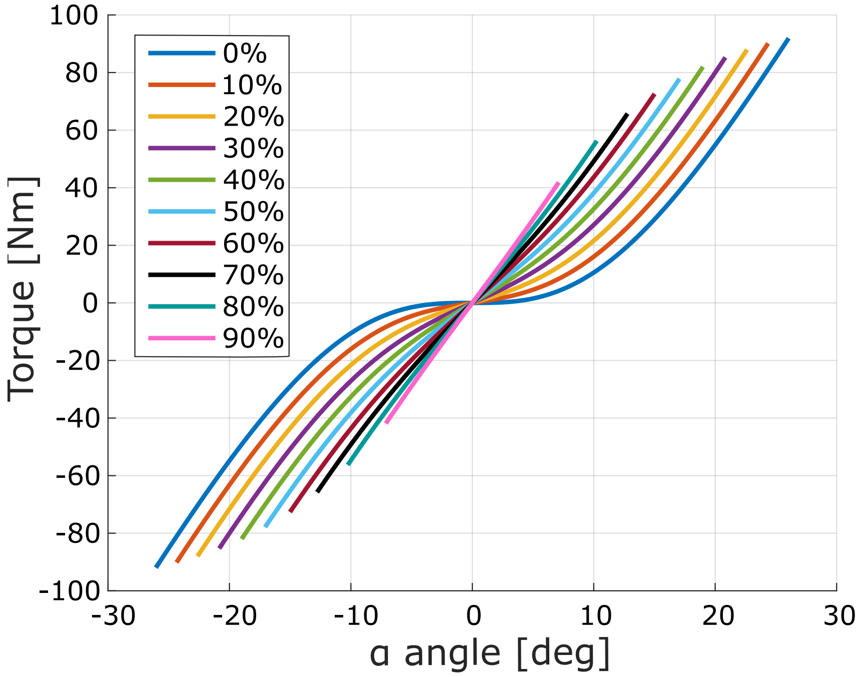

Figure 3: Deflection angle-torque characteristics of the MACCEPA-based ankle actuator used during the experiments with healthy users for different levels of spring pre-compression. Each curve stops at the configuration for which the MACCEPA spring is completely compressed. Changing the spring pre-compression level means modifying the behavior of the actuator from stiffer (with higher levels of spring pre-compression (P) to more compliant (with lower levels of spring pre-compression (P).

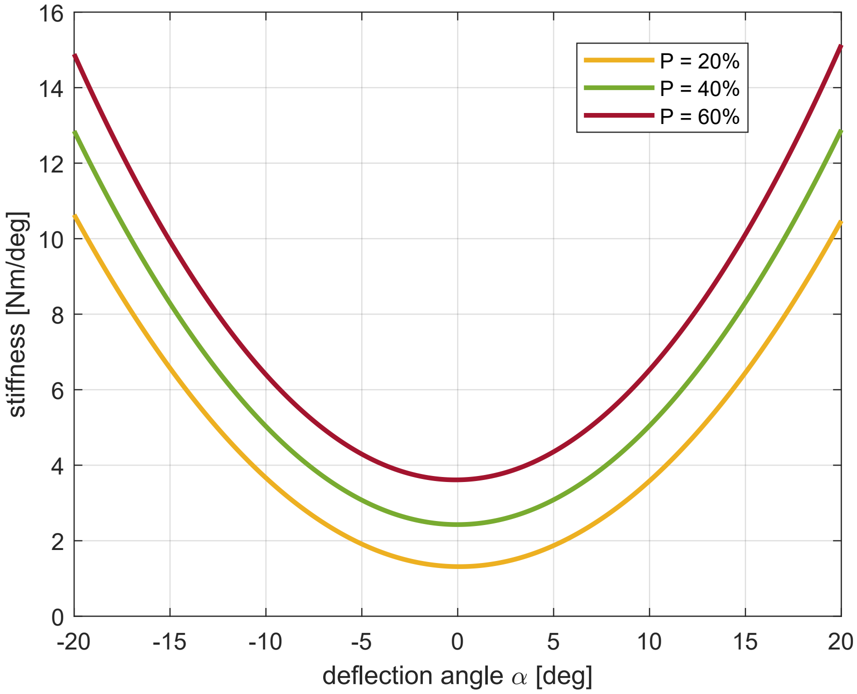

Figure 4: The approximated deflection angle-stiffness characteristics for level of spring pre-compression equal to 20%, 40%, and 60%.
