# Supplementary material for: Walking with a powered ankle-foot orthosis: the effects of actuation timing and stiffness level on healthy users
Source: J Neuroeng Rehabil. 2020 Jul 17;17:98. doi: 10.1186/s12984-020-00723-0 (PMC7367242; doi:10.1186/s12984-020-00723-0)
Supplement: Supplementary file 2 — Additional file 2 Document 2. Algorithm used for heel strike detection. [file 12984_2020_723_MOESM2_ESM.pdf]

Footswitch control algorithm

The initiation of a new step in the actuator's controller needs to be detected by means of a signal coming from a footswitch placed at the heel of the user. To create this signal during the walking trials, a force sensing resistor (SEN-09376 Antratek used with Phidgets Voltage Divider 1121) was attached below the heel plate of the PAFO to detect the contact of the heel with the ground. This force sensing resistor is referred to as footswitch hereafter. The actuator's controller takes as input for the detection of the contact of the heel with the ground a signal with only two possibilities for its value: *high* (equal to 1) and *low* (equal to 0). To transform the raw footswitch signal into the desired on-off signal, the algorithm shown in Fig. 1a was implemented in the controller of the actuator.

This algorithm works with two thresholds. The main idea of the algorithm is explained in the following: if the output value of the algorithm (OnOffHS) is already high (i.e. equal to 1), the foot is on the ground, thus, the output of the algorithm will keep being high unless the raw signal of the footswitch (RawHS) becomes lower than a predefined low threshold (LTh). When this condition is satisfied, OnOffHS is changed to low (i.e. to 0). After this, OnOffHS is kept being low until RawHS becomes higher than a predefined high threshold (UTh). Figure 1b shows the input measured and output generated by the algorithm during one of the walking experiments performed with users. During the experiments, the levels for LTh and UTh were manually modified based on the minimum and maximum input values read by the footswitch during the gait cycle.

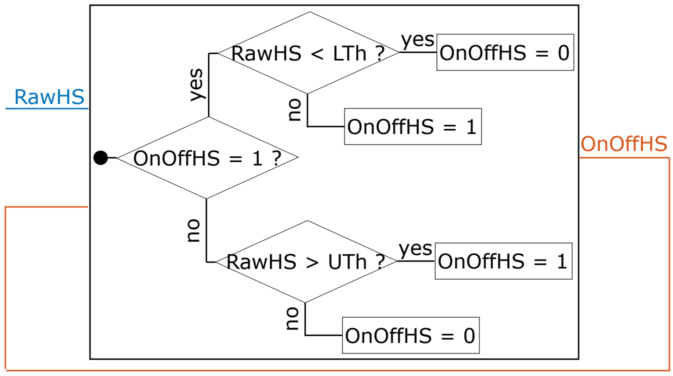

(a) Algorithm

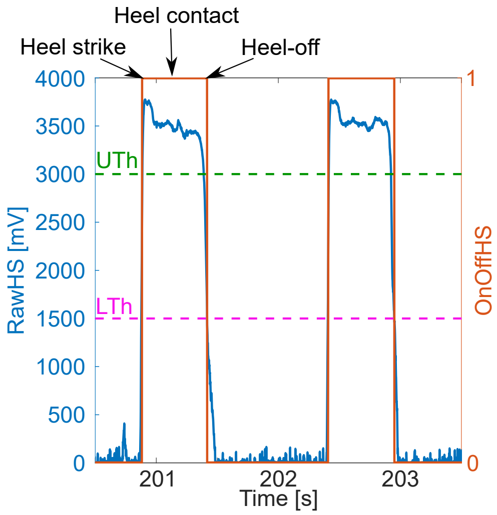

(b) Input (RawHS) and output (OnOffHS) signals of the algorithm

Figure 1: The algorithm to detect the heel contact implemented in the controller of the actuator for the walking trials with users. On the left, the working principle of the algorithm is shown. On the right, an example of the performance of the algorithm is given. The graph shows the raw footswitch signal (RawHS, in blue in both figures) and the output of the algorithm (OnOffHS, in orange in both figures) during one of the walking trials performed with healthy subjects. In this walking trial the thresholds for the algorithm (LTh and UTh) were set as: LTh = 1500 mV and UTh = 3000 mV.
