# Supplementary figures and images for: Walking with a powered ankle-foot orthosis: the effects of actuation timing and stiffness level on healthy users
Source: J Neuroeng Rehabil. 2020 Jul 17;17:98. doi: 10.1186/s12984-020-00723-0 (PMC7367242; doi:10.1186/s12984-020-00723-0)

# MINUTE 1

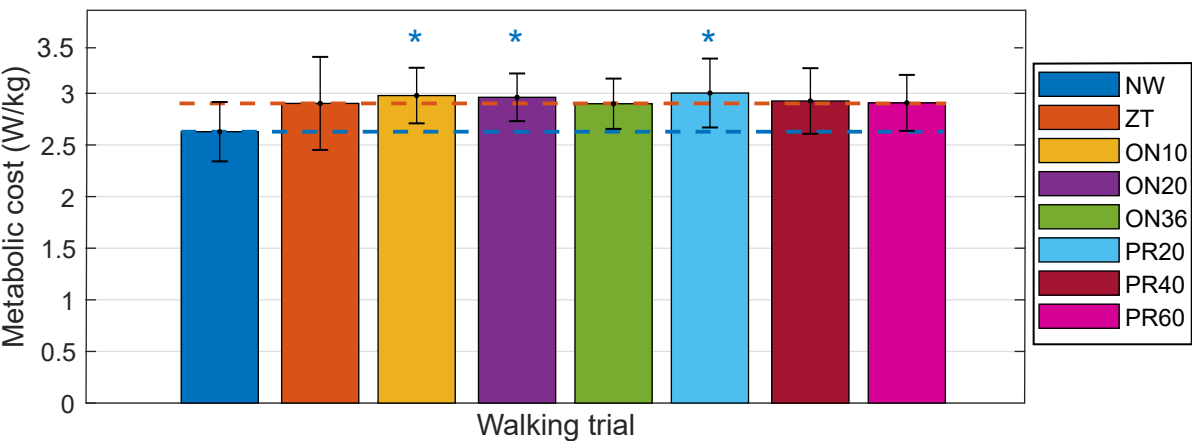

# MINUTE 10

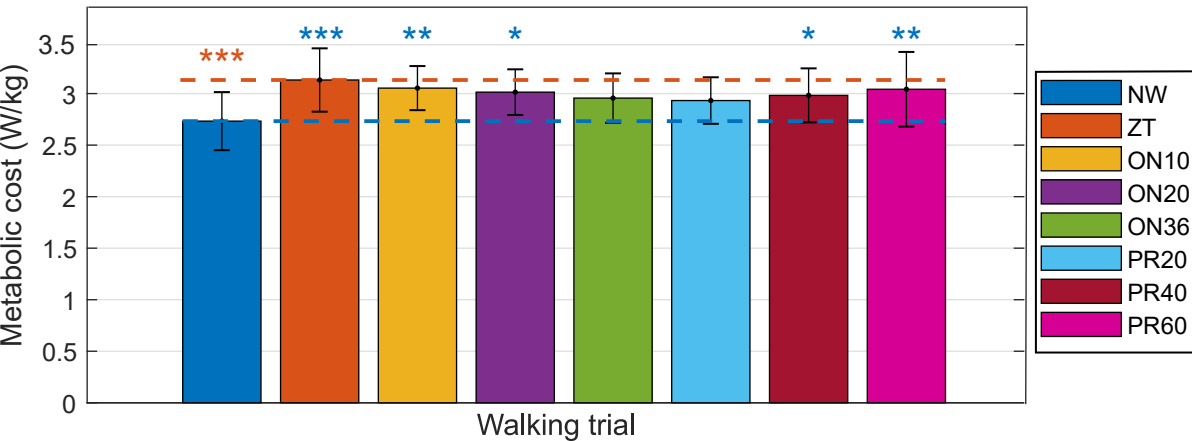

Supplement: Supplementary file 3 — Additional file 3 Figure S1. Net metabolic cost of walking in different walking conditions at the first and last minute of walking. To improve the readability of the graph, the values of the net metabolic costs of normal walking (NW) and zero torque (ZT) are reported across the other conditions with a horizontal dashed line (blue for NW and orange for ZT). Statistically significant difference in the net metabolic cost of the walking trials as compared to NW and ZT is reported with blue and orange asterisks, respectively. * indicates p ≤0.05, ** indicates p ≤0.01, *** indicates p ≤0.001. [file 12984_2020_723_MOESM3_ESM.pdf]

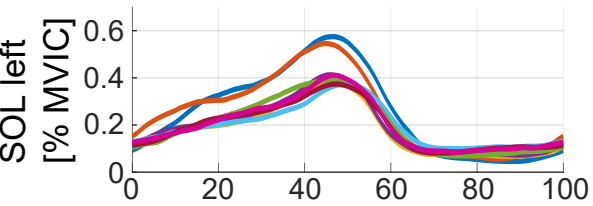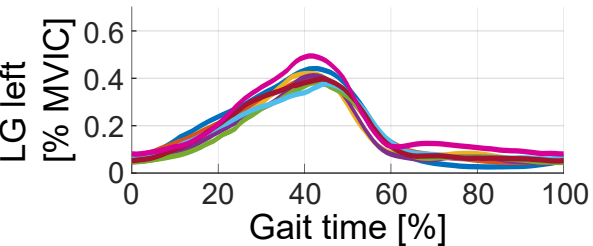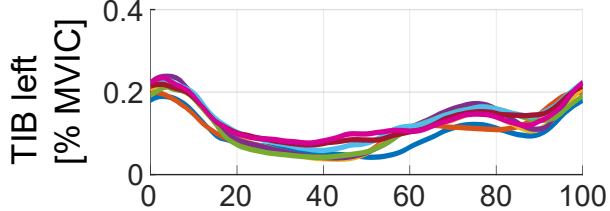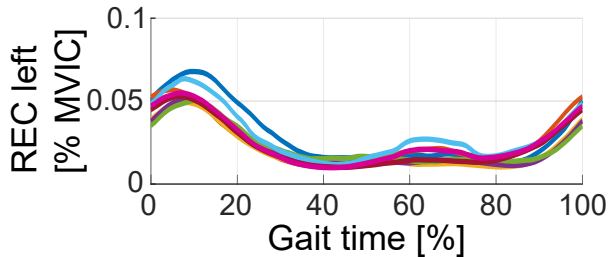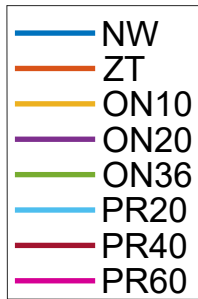

Supplement: Supplementary file 4 — Additional file 4 Figure S2. Envelopes of the soleus (SOL), tibialis anterior (TIB), lateral gastrocnemius (LG), and rectus femoris (REC) activity at the leg wearing the MAPO during different walking conditions at the last minute of walking. The envelopes are normalized such that 1 corresponds to the maximum value obtained during the MVIC exercises and they are the averaged envelopes of the different subjects. [file 12984_2020_723_MOESM4_ESM.pdf]

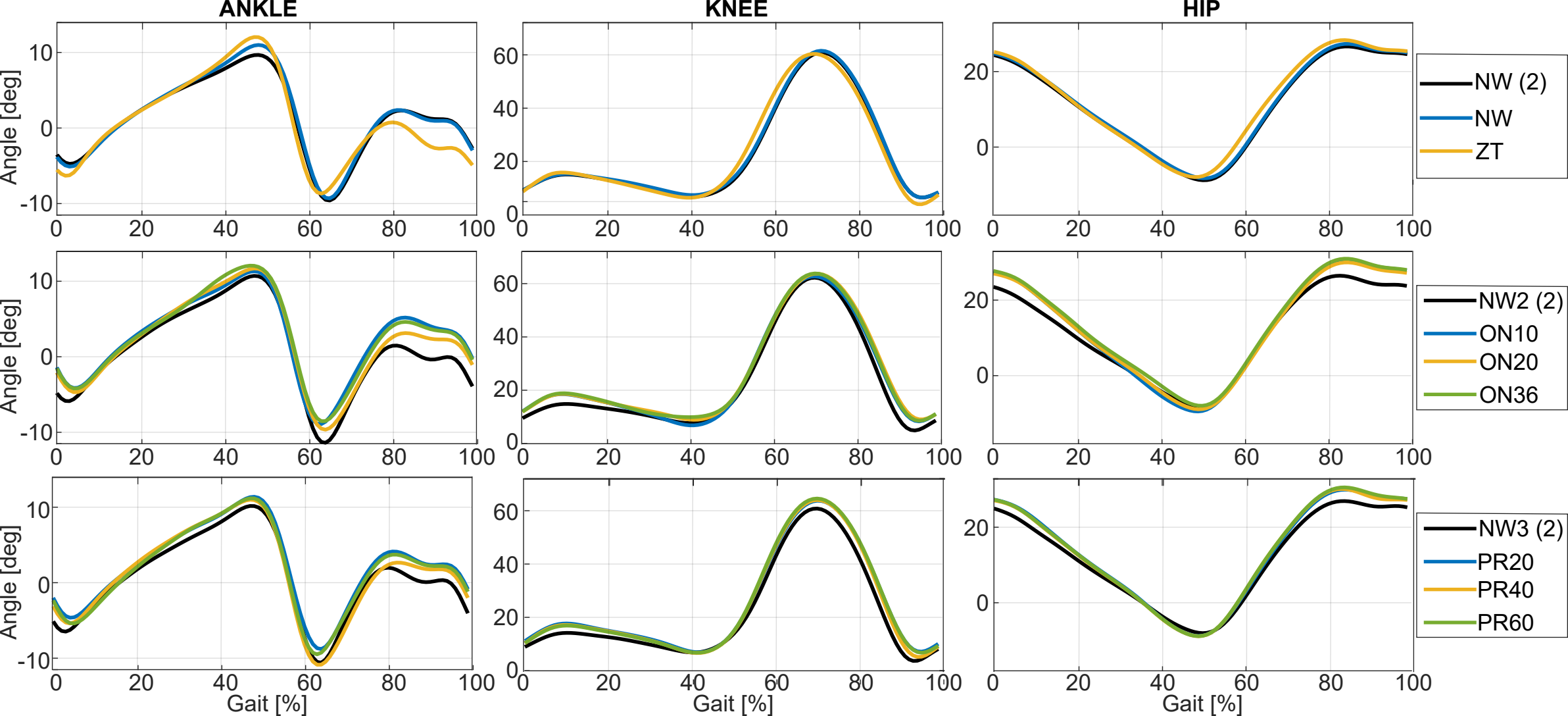

Supplement: Supplementary file 6 — Additional file 6 Figure S3. Hip, knee, and ankle joints kinematics in the sagittal plane at the assisted leg during different walking conditions. The joint trajectories are given for the last minute of walking. For each session (each row), the joints trajectories for the second minute of walking in the NW condition is also given (NW(2), NW2(2), and NW3(2)). [file 12984_2020_723_MOESM6_ESM.pdf]

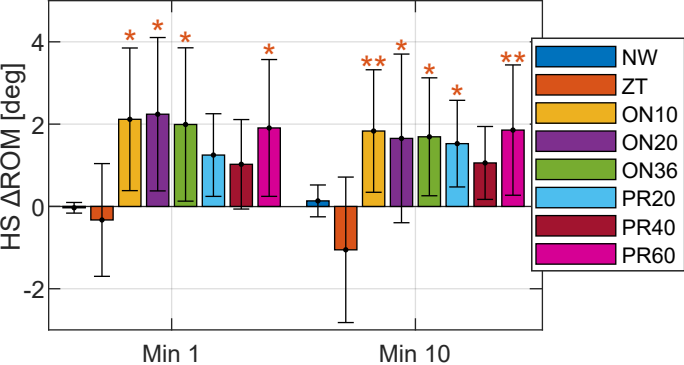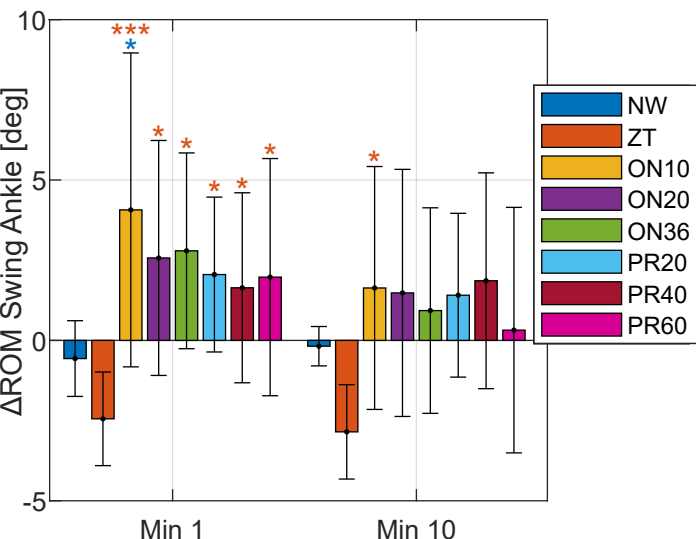

Supplement: Supplementary file 8 — Additional file 8 Figure S4. Left ankle HS ROM and left ankle swing ROM during different walking conditions at the first and last minute of walking. Statistically significant differences as compared to NW and ZT are reported with blue and orange asterisks, respectively. * indicates p ≤0.05, ** indicates p ≤0.01, and *** indicates p ≤0.001. [file 12984_2020_723_MOESM8_ESM.pdf]
