# Supplementary material for: Walking with a powered ankle-foot orthosis: the effects of actuation timing and stiffness level on healthy users
Source: J Neuroeng Rehabil. 2020 Jul 17;17:98. doi: 10.1186/s12984-020-00723-0 (PMC7367242; doi:10.1186/s12984-020-00723-0)
Supplement: Supplementary file 5 — Additional file 5 Table S1. RMS of the activity of the lower limbs muscles across walking conditions at the last minute of walking. [file 12984_2020_723_MOESM5_ESM.pdf]

Condition	Left RMS TIB LR [%MVIC]	Right RMS TIB LR [%MVIC]	Left RMS TIB Swing [%MVIC]	Right RMS TIB Swing [%MVIC]	Left RMS LG Stance [%MVIC]	Right RMS LG Stance [%MVIC]	Left RMS REC Stance [%MVIC]	Right RMS REC Stance [%MVIC]	Left RMS REC Swing [%MVIC]	Right RMS REC Swing [%MVIC]	Left RMS SOL Stance [%MVIC]	Right RMS SOL Stance [%MVIC]
**NW**	0.18±0.07	0.20±0.04	0.12±0.04	0.13±0.04	0.29±0.15	0.22±0.21	0.04±0.02	0.04±0.04	0.02±0.01	0.03±0.03	0.40±0.15	0.39±0.10
**ZT**	0.18±0.07	0.17±0.05	0.14±0.06	0.13±0.06	0.28±0.14	0.23±0.20	0.03±0.02	0.04±0.02	0.02±0.02	0.03±0.02	0.39±0.11	0.37±0.10
**ON10**	0.21±0.07	0.17±0.04	0.14±0.07	0.11±0.03	0.27±0.15	0.24±0.17	0.03±0.02	0.03±0.02	0.02±0.01	0.02±0.01	0.27±0.06	0.41±0.10
**ON20**	0.23±0.08	0.17±0.02	0.16±0.07	0.11±0.03	0.26±0.13	0.23±0.17	0.03±0.02	0.03±0.02	0.02±0.01	0.02±0.01	0.28±0.10	0.42±0.10
**ON36**	0.20±0.06	0.18±0.05	0.16±0.07	0.11±0.03	0.25±0.12	0.24±0.15	0.03±0.02	0.03±0.02	0.02±0.01	0.02±0.01	0.29±0.11	0.41±0.09
**PR20**	0.22±0.09	0.20±0.06	0.16±0.09	0.13±0.04	0.27±0.09	0.20±0.12	0.04±0.03	0.03±0.02	0.03±0.03	0.02±0.02	0.27±0.06	0.42±0.07
**PR40**	0.21±0.09	0.20±0.08	0.16±0.07	0.14±0.04	0.28±0.13	0.23±0.14	0.03±0.02	0.03±0.02	0.02±0.01	0.02±0.01	0.28±0.07	0.43±0.08
**PR60**	0.22±0.11	0.21±0.07	0.15±0.09	0.15±0.04	0.32±0.12	0.24±0.16	0.03±0.02	0.03±0.02	0.03±0.02	0.02±0.01	0.29±0.09	0.43±0.08

Table : RMS of the activity of the lower limbs muscles across walking conditions. Data are reported for the last minute of walking during different walking conditions. The data are given as mean±standard deviation of the data collected in different subjects and they are normalized such that 1 corresponds to the maximum value obtained during the MVIC exercises. The RMS activity of different muscles is calculated in relevant sub-phases of the gait cycle, i.e. the loading response (LR), the stance, and the swing sub-phases.
