# Supplementary material for: Walking with a powered ankle-foot orthosis: the effects of actuation timing and stiffness level on healthy users
Source: J Neuroeng Rehabil. 2020 Jul 17;17:98. doi: 10.1186/s12984-020-00723-0 (PMC7367242; doi:10.1186/s12984-020-00723-0)
Supplement: Supplementary file 7 — Additional file 7 Table S2. Kinematics parameters measured in different walking conditions at the last minute of walking. [file 12984_2020_723_MOESM7_ESM.pdf]

Condition	LEFT Ankle HS ΔROM [deg]	LEFT Ankle Swing ΔROM [deg]	LEFT Ankle Stance ΔROM [deg]	LEFT Knee ΔROM [deg]	LEFT Hip ΔROM [deg]	RIGHT Ankle HS ΔROM [deg]	RIGHT Ankle Swing ΔROM [deg]	RIGHT Ankle Stance ΔROM [deg]	RIGHT Knee ΔROM [deg]	RIGHT Hip ΔROM [deg]
**NW**	0.13±0.39	-0.18±0.61	0.98±1.21	0.04±0.99	0.14±1.28	0.23±0.37	-0.45±0.98	0.71±1.43	0.22±1.85	0.08±1.13
**ZT**	-1.05±1.77	-2.85±1.47	2.03±1.74	0.51±2.45	0.97±1.98	-0.07±1.57	-0.98±2.15	0.17±2.91	0.92±2.14	0.02±1.58
**ON10**	1.83±1.49	1.63±3.79	-0.54±3.20	-1.24±4.20	5.26±2.53	0.28±0.51	0.52±2.29	-0.21±2.26	-0.24±1.83	1.73±1.67
**ON20**	1.65±2.05	1.48±3.85	0.60±2.61	-0.67±4.61	5.42±2.10	0.90±1.00	-0.58±4.67	0.79±1.96	0.42±1.78	1.73±2.08
**ON36**	1.69±1.43	0.93±3.20	-0.58±4.86	-0.86±4.26	4.91±3.21	0.73±0.94	-0.38±2.77	-0.58±2.36	-0.03±2.03	2.29±2.26
**PR20**	1.53±1.05	1.41±2.55	0.44±2.08	0.92±3.57	4.51±2.71	0.74±1.07	-0.53±2.18	0.38±1.46	0.94±3.25	2.58±2.58
**PR40**	1.06±0.89	1.86±3.36	2.09±2.22	2.56±4.13	4.14±3.00	0.26±0.76	-0.46±2.40	0.86±1.91	1.27±2.47	2.06±2.95
**PR60**	1.86±1.58	0.32±3.83	1.07±1.98	1.95±4.35	4.18±2.96	0.49±1.14	-0.71±2.43	0.39±2.09	0.57±2.70	2.04±2.98

Table : Kinematics parameters measured in different walking conditions. The data are reported as mean ± standard deviation of the data collected in different subjects and they are given for the last minute of walking (minute 10). For each walking trial, the ΔROM is calculated as the ROM measured during the walking trial minus the ROM measured during the normal walking trial of the same session (i.e. NW for NW and ZT, NW2 for ON10, ON20, and ON36, NW3 for PR20, PR40, and PR60) at minute 2.
