# Supplementary material for: Walking with a powered ankle-foot orthosis: the effects of actuation timing and stiffness level on healthy users
Source: J Neuroeng Rehabil. 2020 Jul 17;17:98. doi: 10.1186/s12984-020-00723-0 (PMC7367242; doi:10.1186/s12984-020-00723-0)
Supplement: Supplementary file 9 — Additional file 9 Table S3. Gait parameters measured in different walking conditions at the last minute of walking. [file 12984_2020_723_MOESM9_ESM.pdf]

Condition	Stride width [cm]	LEFT Stance Perc [% gait]	RIGHT Stance Perc [% gait]	LEFT Stride Length [m]	RIGHT Stride Length [m]
**NW**	16.43±2.47	64.46±1.06	65.40±1.23	1.10±0.12	1.10±0.12
**ZT**	16.44±2.03	62.89±1.20	65.94±1.09	1.10±0.14	1.10±0.14
**ON10**	16.43±2.53	62.51±1.15	66.01±1.19	1.16±0.11	1.16±0.11
**ON20**	15.99±2.16	62.66±1.46	67.13±2.76	1.15±0.11	1.15±0.11
**ON36**	16.36±1.63	62.98±1.35	65.81±1.24	1.15±0.10	1.15±0.11
**PR20**	17.39±2.31	63.14±1.28	66.38±1.68	1.15±0.13	1.15±0.12
**PR40**	16.79±2.46	62.96±0.88	66.23±1.11	1.14±0.11	1.14±0.11
**PR60**	16.89±2.26	62.40±0.99	66.06±1.19	1.15±0.10	1.15±0.10

Table : Gait parameters measured in different walking conditions. The data are reported as mean ± standard deviation of the data collected in different subjects and they are given for the last minute of walking (minute 10).
